# Genome-wide association analysis permits characterization of Stagonospora nodorum blotch (SNB) resistance in hard winter wheat

**DOI:** 10.1038/s41598-021-91515-6

**Published:** 2021-06-15

**Authors:** Rami AlTameemi, Harsimardeep S. Gill, Shaukat Ali, Girma Ayana, Jyotirmoy Halder, Jagdeep S. Sidhu, Upinder S. Gill, Brent Turnipseed, Jose L. Gonzalez Hernandez, Sunish K. Sehgal

**Affiliations:** 1 Department of Agronomy, Horticulture and Plant Science, South Dakota State University, Brookings, SD 57007 USA; 2 Department of Plant Pathology, North Dakota State University, Fargo, ND 58108 USA

**Keywords:** Plant breeding, Plant genetics, Agricultural genetics, Plant breeding, Plant genetics, Quantitative trait

## Abstract

Stagonospora nodorum blotch (SNB) is an economically important wheat disease caused by the necrotrophic fungus *Parastagonospora nodorum*. SNB resistance in wheat is controlled by several quantitative trait loci (QTLs). Thus, identifying novel resistance/susceptibility QTLs is crucial for continuous improvement of the SNB resistance. Here, the hard winter wheat association mapping panel (HWWAMP) comprising accessions from breeding programs in the Great Plains region of the US, was evaluated for SNB resistance and necrotrophic effectors (NEs) sensitivity at the seedling stage. A genome-wide association study (GWAS) was performed to identify single‐nucleotide polymorphism (SNP) markers associated with SNB resistance and effectors sensitivity. We found seven significant associations for SNB resistance/susceptibility distributed over chromosomes 1B, 2AL, 2DS, 4AL, 5BL, 6BS, and 7AL. Two new QTLs for SNB resistance/susceptibility at the seedling stage were identified on chromosomes 6BS and 7AL, whereas five QTLs previously reported in diverse germplasms were validated. Allele stacking analysis at seven QTLs explained the additive and complex nature of SNB resistance. We identified accessions (‘Pioneer-2180’ and ‘Shocker’) with favorable alleles at five of the seven identified loci, exhibiting a high level of resistance against SNB. Further, GWAS for sensitivity to NEs uncovered significant associations for SnToxA and SnTox3, co-locating with previously identified host sensitivity genes (*Tsn1* and *Snn3*). Candidate region analysis for SNB resistance revealed 35 genes of putative interest with plant defense response-related functions. The QTLs identified and validated in this study could be easily employed in breeding programs using the associated markers to enhance the SNB resistance in hard winter wheat.

## Introduction

Wheat (*Triticum aestivum L*.) is the largest grown cereal crop in the world and plays a crucial role in human food supply^1^. Wheat demand is expected to surge by 60% to feed the projected population of 9 billion by 2050^2^. However, wheat productivity is continuously constrained by biotic and abiotic factors, including fungal diseases^3^. Globally, these fungal diseases comprise wheat rusts, blights, and leaf spot diseases, including Stagonospora nodorum blotch (SNB). SNB, caused by a necrotrophic fungus *Parastagonospora nodorum* (*Berk.*) {syn. *Septoria nodorum*, *Stagonospora nodorum*; teleomorph *Phaeosphaeria nodorum*), is an important disease in most wheat-growing regions of the world^4,5^. The disease is common in Australia, the US, and parts of northern Europe, causing significant yield losses^4,6–8^. In the US, SNB is a recurrent disease of wheat in several geographic regions, including the Pacific Northwest, the upper Great Plains, and the Eastern states^7^. Adoption of no- or minimum tillage practices may have further increased the incidence of disease in winter-wheat growing regions of the US. Fungicides are generally used to control SNB; however, there have been several reports where high selection pressure among the pathogen populations has led to the development of resistance in the pathogen against several fungicides^9,10^. Thus, breeding for genetic resistance against SNB with reduced dependency on fungicides is a durable and environmental-friendly approach to manage SNB in wheat.

The biotrophic pathogens require living tissue and establish a long-term plant-pathogenic feeding relationship. To combat the biotrophic pathogens, plants have innate immune systems that activate the pathogen-associated molecular pattern (PAMP)-triggered immunity (PTI) and effector-triggered immunity (ETI) pathways^11^, which leads to the resistance following a classical gene-for-gene hypothesis^12^. By contrast, *Parastagonospora nodorum* is a necrotrophic pathogen, and its host interaction follows an inverse gene-for-gene model^13^. In this case, the pathogen secretes proteins known as necrotrophic effectors (NEs) that interact with corresponding host sensitivity loci (*Snn*) and cause programmed cell death^14^. The first NE (PtrToxA) triggered susceptibility was observed in the wheat-*Pyrenophora tritici-repentis* pathosystem that causes tan spot in wheat germplasm carrying sensitivity gene *Tsn1*^15,16^. A nearly identical NE (SnToxA) was identified in *Parastagonospora nodorum*^17^ with a corresponding host sensitivity gene, *Tsn1*. Compared to other NEs present in *P. nodorum*, SnToxA became an important virulence factor, which is believed to be horizontally transferred to *Pyrenophora tritici-repentis*^18^ and *Bipolaris sorokiniana*^19^. In addition to SnToxA, there are several other NEs namely, SnTox1, SnTox2*,* SnTox3, SnTox4, SnTox5, SnTox6, and SnTox7, which interact with their corresponding *Snn* genes present in wheat (*Snn1, Snn2, Snn3, Snn4, Snn5, Snn6,* and *Snn7,* respectively)^13,20–25^. Thus, SNB resistance in wheat largely depends on the presence of these susceptibility genes and is quantitatively inherited^20^.

Linkage analyses based on bi-parental populations have been useful in dissecting the genetic control of SNB resistance. This approach has identified several QTLs for response to SNB on different wheat chromosomes^13,17,20,23,24,26–30^. These QTLs are a valuable resource for breeders to develop SNB resistant cultivars. However, linkage mapping can only encompass the allelic diversity segregating between the parents of the bi-parental population, limiting the scope of this approach^31,32^.

Genome-wide association studies (GWAS) or linkage disequilibrium-based mapping is another approach for dissecting the genetics of complex traits, which overcomes the major limitations of linkage mapping. GWAS involves evaluating marker-trait associations (MTAs) in a large panel of unrelated individuals, harnessing a large number of historical recombinations^33^. GWAS have successfully identified several QTLs affecting yield, quality, biotic- and abiotic- stresses in wheat^32,34–37^. Several GWA studies in wheat identified several QTLs for SNB resistance distributed over chromosomes 1A, 1B, 2A, 2D, 3A, 3B, 4A, 4B, 5A, 5B, 5D, 6A, 6B, 7A, and 7D^32,36,38–42^. These studies employed association-mapping panels comprising a large number of wheat landraces^38,39^, a set of modern cultivars^40,42^, and a historical set of wheat lines^41^; however, most of these studies did not explore the US hard winter wheat cultivars/breeding materials.

In this study, we used a set of 274 accessions from the hard winter wheat association-mapping panel (HWWAMP)^43^ to dissect the complex response to SNB in hard winter wheat. The HWWAMP has been successfully used in several GWA studies^34,35,43,44^ to identify QTLs for disease resistance, grain quality traits, and coleoptile length. We screened the collection for resistance against SNB and sensitivity against SnToxA, SnTox1, and SnTox3. The objectives of the study were (i) to identify and evaluate the genetic basis of resistance against SNB; (ii) identify SNP markers associated with sensitivity to SnToxA, SnTox1, and SnTox3; (iii) identify candidate genes in the regions associated with SNB response.

## Results

### The response of HWWAMP accessions to SNB

**Figure 1:**
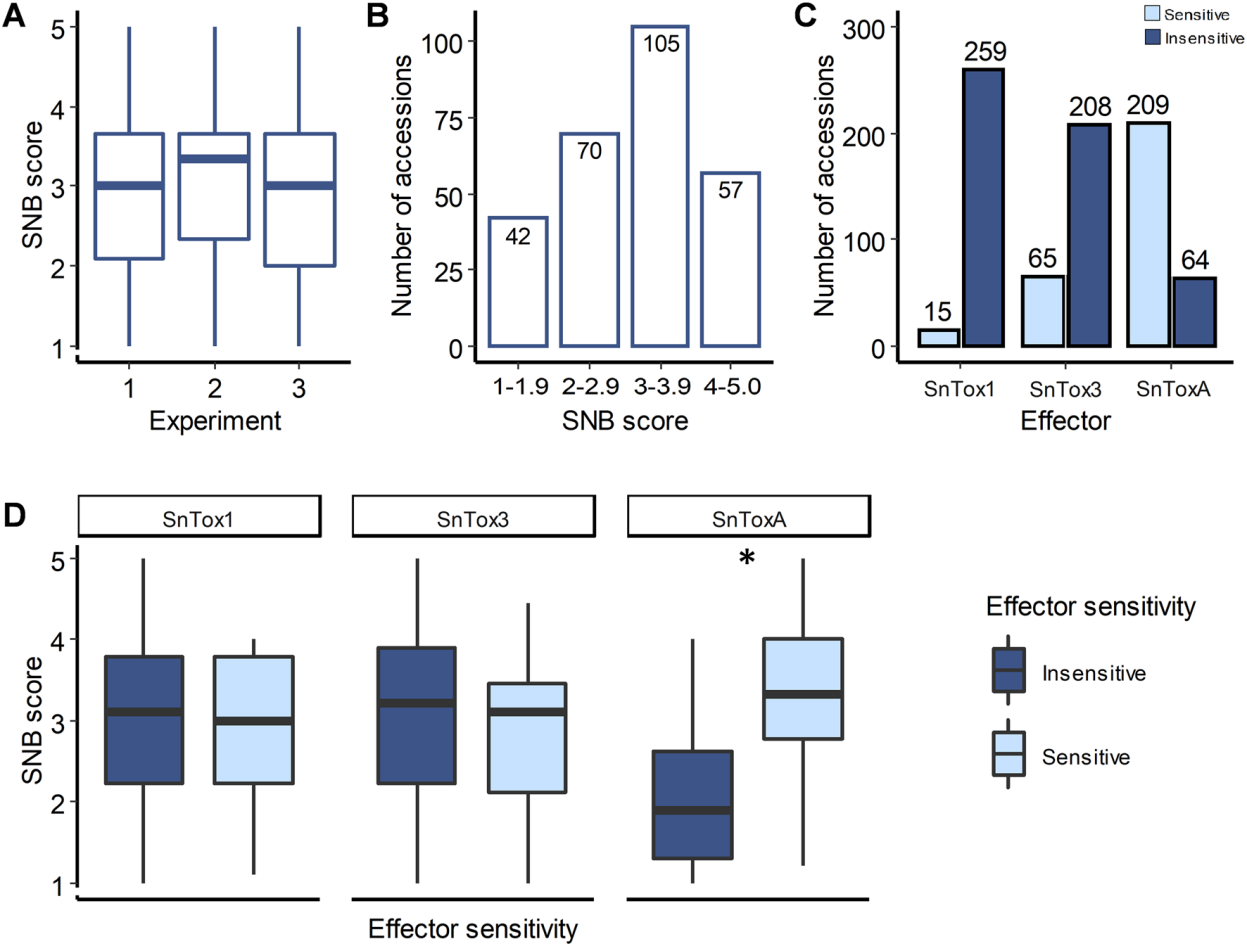
SNB response and necrotrophic-effectors sensitivity reaction of hard winter wheat association mapping panel (HWWAMP) accessions. **(A)** Boxplots showing the distribution of SNB scores of 274 HWWAMP accessions in the three experiments. **(B)** Disease distribution of Sn2000 inoculations in 274 accessions. **(C)** Sensitivity reaction of the 274 accessions against SnToxA*,* SnTox1, and SnTox3. **(D)** Boxplots for average SNB scores versus sensitivity reaction for three necrotrophic effectors. T-test was used for comparison between groups for SNB score. Asterisk denotes significant difference (*P* < *0.01*).

SNB resistance in 274 accessions of HWWAMP was evaluated at the seedling stage in three independent experiments (Exp 1, Exp 2, and Exp 3). These accessions exhibited a wide variation in response to SNB inoculations from highly resistant to fully susceptible genotypes (Fig. 1A). The three experiments were statistically consistent based on the linear mixed model (LMM) analysis. Furthermore, a high correlation (r > 0.90) was observed among the three experiments. The overall mean and median disease scores for SNB infection were 2.95 and 3.00, respectively. The majority of the accessions (105 out of 274) had a disease score within a range of 3.0–3.9, indicating a moderately susceptible response, followed by 70 accessions with a disease score between 2.0 and 2.9 (moderately resistant). Moreover, 57 accessions showed a susceptible response with a disease score of 4.0–5.0, and 42 accessions had a disease score of less than 2, indicating a resistant response (Fig. 1B). Among the resistant lines, seven accessions were having a mean disease score of ‘1’ across all three experiments (Table 1). Four of these seven lines (‘Pioneer-2180’, ‘Colt’, ‘Sturdy-2-K’, and ‘TAM304’) are varieties released after the 1980s from different breeding programs. The grand mean of SNB response for 274 HWWAMP accessions recorded over three experiments is provided in Supplementary Table S1.

**Table 1:** Hard winter wheat association mapping panel (HWWAMP) accessions showing a high level of resistance against SNB, along with their mean disease score across three experiments.

Accession	Year of release	Origin	Pedigree	Disease score	Number of ‘R’ alleles^a^
Pioneer-2180	1989	KS	TAM-101 / Pioneer W603 // Pioneer W558	1.0	5
COLT	1983	NE	Agate sib (NE69441)// (Tx65A1503-1) 391-56-D8/Kaw	1.0	4
E2041		MI	Pioneer 2552/Pioneer 2737W	1.0	3
OK09634		OK	OK95616-98-6756/Overley	1.0	4
SD05210		SD	SD98444/SD97060	1.0	3
STURDY-2-K	2005	TX	Sinvalocho/Wichita//Hope/Cheyenne/3/2* Wichita/4/Seu Seun 27	1.0	3
TAM304	2009	TX	TX92U3060/TX91D6564	1.0	4
NEKOTA	1994	NE	Bennett/TAM 107	1.1	3
OK05723W		OK	SWM866442/Betty	1.1	3

^a^The number of resistance-associated alleles at seven of the MTAs identified in this study.

### Effector sensitivity and SNB response

In addition to SNB infection, we evaluated all 274 accessions for necrotrophic effector sensitivity. Three effector toxins namely SnToxA, SnTox1, and SnTox3 were used to infiltrate all the accessions in independent experiments. For SnToxA, 209 accessions were sensitive and 64 were insensitive. For SnTox3, there were 65 sensitive and 208 insensitive accessions. We did not find sufficient variation in the case of SnTox1 as 259 accessions were insensitive and only 15 accessions were sensitive (Fig. 1C). Furthermore, we determined the contribution of effector sensitivity to the SNB infection by comparing the inoculation and infiltration data. We found a significant difference for SNB disease severity among SnToxA sensitive and insensitive groups (*P* < 2.2e−16) at *P* < 0.01 level of significance (Fig. 1D). The mean SNB score were 1.99 and 3.26 for insensitive and sensitive groups, respectively, indicating that SnToxA-sensitive accessions were significantly more susceptible than SnToxA-insensitive accessions. Contrary to SnToxA, we did not find significant differences among sensitive and insensitive groups for SnTox1. The mean SNB score was 2.95 and 2.90 for SnTox1 insensitive and sensitive groups, respectively. As the isolate Sn2000 lacks SnTox3, the mean SNB score was similar for insensitive and sensitive groups (3.00 and 2.86) as expected (Fig. 1D).

### Population structure and LD analysis

Before performing GWAS, we inferred the population structure among the 274 accessions based on model-based Bayesian clustering in STRUCTURE using 15,590 SNP markers. Population structure analysis revealed three subpopulations (P1, P2, and P3 for later reference) within the 274 accessions based on DeltaK statistic. The three subpopulations, i.e., P1, P2, and P3 consisted of 81, 152, and 41 accessions, respectively (Supplementary Fig. S1). We attempted to determine the relationship between these subpopulations and the breeding program from which these accessions originated. Most of the accessions originating from South Dakota and Nebraska, and all the accessions from Montana fits in subpopulation P2. The accessions from the Colorado breeding program dominated subpopulations P1 and P2, with no accession in P3. Contrary to this, the accessions from Kansas, Oklahoma, and Texas were evenly distributed among all three subgroups. The mean SNB score for P1, P2, and P3 was 3.05, 2.98, and 2.60, respectively, indicating that accessions from P1 and P2 incline towards moderately susceptible reaction and P3 being moderately resistant.

Linkage Disequilibrium (LD) analysis for HWWAMP accessions has already been performed using the same set of SNP markers in our previous study^35^. We estimated the LD decay based on the r^2^ values for the whole genome and individual genomes. The distance where LD value (r^2^) decreases below 0.1 or half strength of D' (D' = 0.5) was estimated based on the curve of the nonlinear logarithmic trend line. LD decay was estimated to be 4.5 cM for the whole genome, whereas LD decay was around 3.4, 3.6 cM, and 14.2 cM in A, B, and D genomes, respectively.

### GWAS for SNB and necrotrophic effectors (NEs)

Association analysis was performed in hard winter wheat panel for SNB and effector (NE) sensitivity, and MTAs were identified for respective phenotypes. Two different algorithms, namely MLM and FarmCPU were initially compared to select the best algorithm for association analysis. The best algorithm was selected for each trait by comparing the model fitness by analyzing the QQ plots (Supplementary Fig. S2). FarmCPU better fit the SNB response, while MLM was selected for the SnToxA, SnTox1, and SnTox3 infiltrations. The best model was used to report significant MTAs for each trait based on a genome-wide significance threshold of *P* < 2.34 × 10^–6^ (− log_10_ *P* > 5.50) after Bonferroni correction of *P*-values, which is highly conservative and reduces the type I errors.

**Figure 2:**
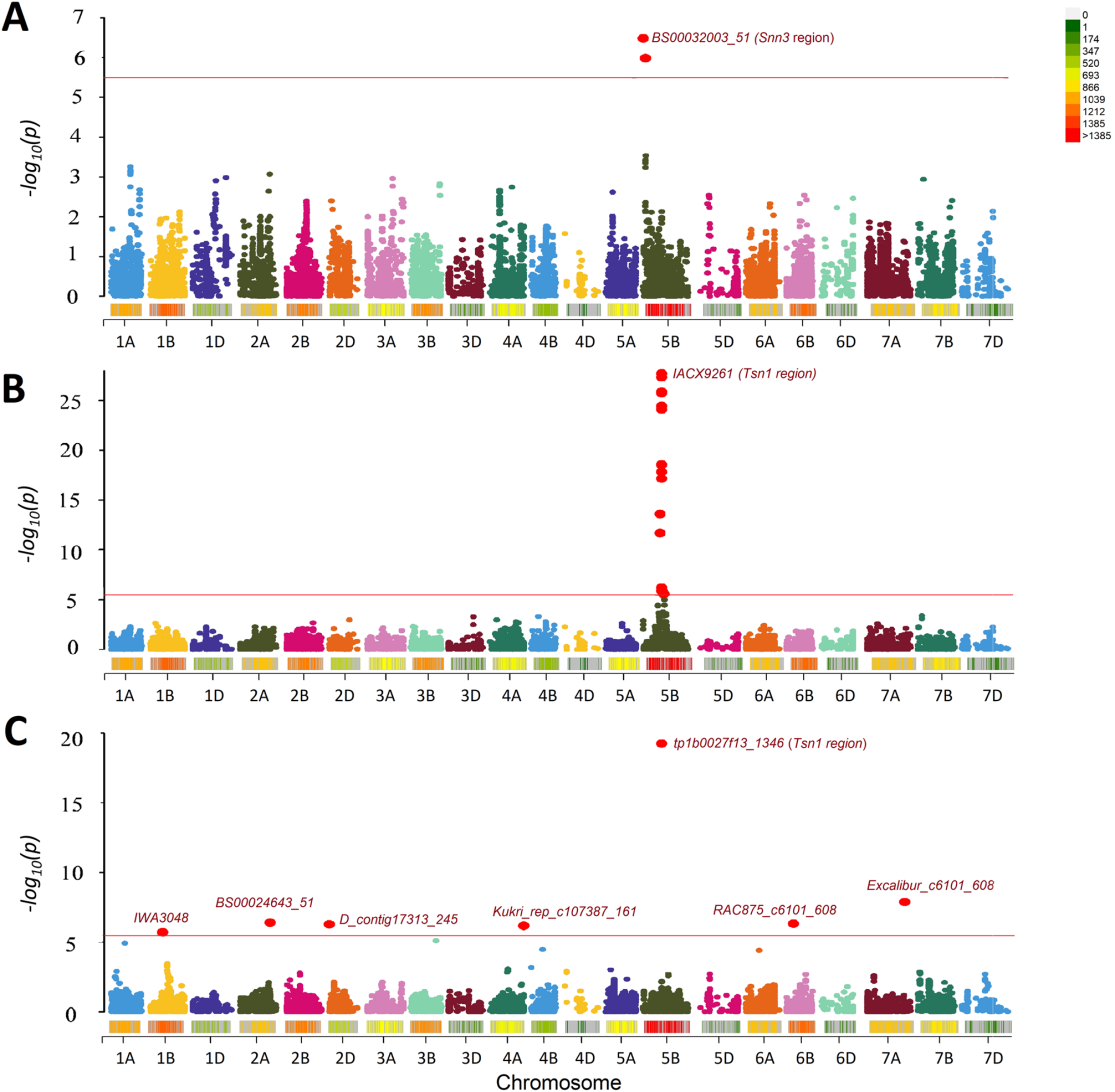
A Manhattan plot representing the marker-trait associations (MTAs) identified for SNB response and NEs reactions. **(A)** The MTAs for SnTox3 infiltrations, **(B)** MTAs for SnToxA infiltrations; **(C)** and MTAs for SNB response. The color scale indicates SNP density on the bar given in the inset. The red line depicts the Bonferroni corrected threshold for identifying significant associations. The significant associations are represented with red dots.

GWAS for SNB response identified a total of seven significant MTAs for SNB resistance/susceptibility (Table 2; Fig. 2). The seven MTAs, representing seven distinct QTLs, were distributed on chromosomes 1B, 2AL, 2DS, 4AL, 5BL, 6BS, and 7AL (Table 2). The QTL (*QSnb.sdsu-5B*) with the largest effect was detected on chromosome 5BL, which corresponds to the genomic location of the susceptibility locus *Tsn1*. The most significant SNP (*tplb0027f13_1346*) for this association was physically mapped to 546 Mb on chromosome 5B (IWGSC RefSeq v1.1), which co-localized with the location of *Tsn1*. The second most significant association was detected near the distal end of the long arm of chromosome 7A, which seems to be a robust QTL (*QSnb.sdsu-7A*) imparting SNB resistance/susceptibility (Table 2). The most significant SNP (*Excalibur_c6101_608*) for this association showed high significance (-log_10_*P* = 7.89) and a marker effect of 0.39. Apart from these, five more associations were declared significant after Bonferroni corrected *p*-values. Another MTA identified on the chromosome 2DS, which mapped at 9 Mb on the physical map (IWGSC RefSeq v1.1), co-localizes with the known locus *Snn2* (6–12 Mb). All the significant associations are enlisted in Table 2, along with their physical location and corresponding marker effect.

**Table 2:** Summary of the significant markers associated with SNB resistance and necrotrophic-effector sensitivity.

Trait	SNP^a^	Allele	Chromosome	Position^b^	effect	*P*-value	FDR Adj (*P-*value)	− Log_10_(*P*)
SNB	*IWA3048*	G/A	1B	364,419,320	0.2252	1.94e−06	0.0043	5.7124
SNB	*BS00024643_51*	C/T	2AL	779,207,329	− 0.1953	3.91e−07	0.0016	6.4080
SNB	*D_contig17313_245*	C/A	2DS	9,343,858	0.2222	5.13e−07	0.0016	6.2897
SNB	*Kukri_rep_c107387_161*	A/G	4AL	742,034,488	− 0.2928	6.59e−07	0.0017	6.1811
SNB	*tplb0027f13_1346*	C/T	5BL	546,827,934	− 0.4680	5.60e−20	8.74e−16	19.2514
SNB	*RAC875_c55270_272*	A/G	6BS	30,036,111	0.1908	4.61e−07	0.0016	6.3362
SNB	*Excalibur_c6101_608*	T/C	7AL	721,174,878	0.3999	1.31e−08	0.0001	7.8819
SnToxA	*IACX9261*	T/G	5BL	546,704,036	0.4079	1.41e−29	2.19e−25	28.8500
SnTox3	*BS00032003_51*	G/A	5BS	2,559,360	− 0.1674	1.72e−07	0.0026	6.7700

All the markers were declared significant based on Bonferroni corrected significance threshold value of − log_10_ *P* > 5.50.

^a^SNP markers are from Infinium 90K  array-based SNPs (Wang et al. 2015).

^b^Physical location is based on IWGSC RefSeq v 1.1 (2018).

**Figure 3:**
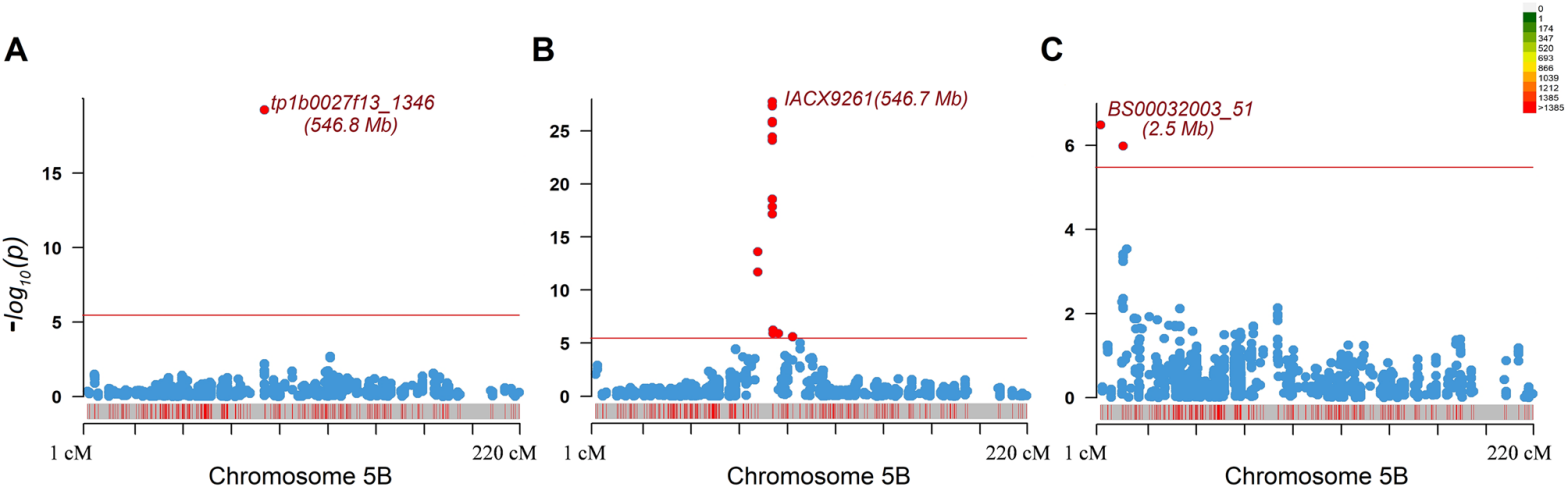
Marker-trait associations detected on chromosome 5B for **(A)** SNB inoculations, **(B)** SnToxA infiltrations, and **(C)** SnTox3 infiltrations. The color scale indicates SNP density on the bar given in the inset. The red line depicts the Bonferroni corrected threshold to declare significant associations. The physical location for the most significant SNP has been provided based on IWGSC RefSeq v 1.1 (2018).

In addition to SNB response, MTAs were detected for SnToxA and SnTox3 infiltrations based on Bonferroni corrected genome-wide significance threshold. A single genomic region was identified on the long arm of chromosome 5B for SnToxA, which again co-locates with the genomic region of *Tsn1*. The physical location of the most significant SNP for SnToxA is the same as that detected on chromosome 5BL for SNB inoculations (Table 2). Furthermore, a significant association was identified for SnTox3 sensitivity on the short arm of chromosome 5B. We also compared the physical locations of MTAs detected chromosome 5B for SNB response, SnToxA, and SnTox3 (Fig. 3). The association for SnTox3 mapped around the 3 Mb region on the chromosome 5B in the physical map, whereas the SnToxA has mapped around 546 Mb (IWGSC RefSeq v1.1). The SnTox3 associated region in our study corresponded to the reported physical location of the *Snn3* gene, corroborating several other reports for association in this region (Table 2; Fig. 2). Contrary to SnToxA and SnTox3, no significant association was detected in the case of SnTox1 infiltrations, owing to the low variation for SnTox1 sensitivity among HWWAMP accessions.

### Allele stacking analysis

**Figure 4:**
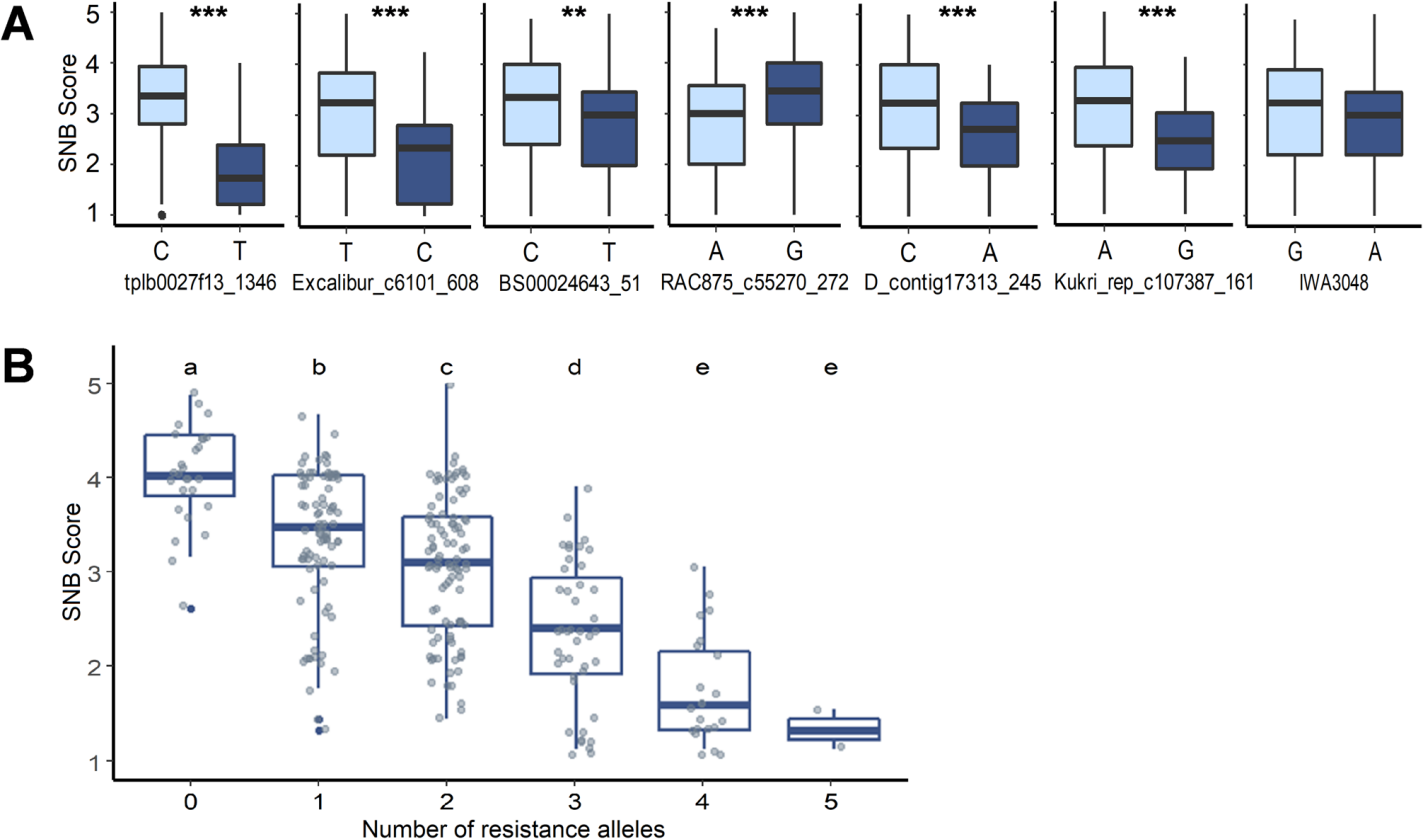
(**A)** Pairwise comparison for SNB score among two alleles of the seven significant MTAs identified for SNB resistance on chromosomes 5BL, 7AL, 2AL, 6BS, 2DS, 4AL, and 1B, respectively (enlisted in Table 2). A t-test was used to compare the two groups. Two asterisks denote significant difference at (*P* < *0.01*) and three asterisks denote significant difference at (*P* < *0.001*). **(B)** Effect of accumulation of resistant alleles for the detected associations on SNB disease score. The different groups were compared using FDR corrected pairwise t-test. Levels denoted by different letters are significantly different (*P* < *0.05*).

The nature of SNB resistance is complex and governed by several active NE-receptor interactions which could vary in different environments. Thus, we studied the effect of accumulation of resistant alleles at seven of the detected QTLs, including *Tsn1* and *Snn2*. Different accessions from the HWWAMP were grouped based on the number of resistant alleles they carry for these seven QTLs. Although seven MTAs were detected in GWAS for SNB response, only six groups of accessions were identified in total, carrying zero to five resistance alleles (Fig. 4). None of the accessions of HWWAMP carry all seven favorable alleles. The mean and median SNB scores for the accessions (group 0) carrying no resistance allele were 4.11 and 4.00, respectively. On the other hand, group 5 comprising two accessions and having resistant alleles at five of the seven loci showed a mean and median SNB score of 1.22 and 1.20, respectively. Similarly, the group of accessions (group 4) with four resistant alleles had a mean and median SNB score of 1.66 and 1.50, respectively. Furthermore, these groups (group 0–group 5) were compared using FDR corrected pairwise t-test to verify the additive effect of the resistant alleles on SNB reaction. The differences in mean SNB scores were statistically significant and the accessions that carried a higher number of resistant alleles were having the lower mean SNB scores and vice versa.

### Exploring the candidate genes

We used five out of the seven MTAs (except the 5BL region corresponding to *Tsn1* and a potential association on chromosome 1B) identified in GWAS for SNB to explore the putative candidate genes. For each MTA, a two Mb window was used to identify the candidate genes. In total, we identified 166 High Confidence genes for the five MTAs based on CS RefSeq 1.1. The functional annotation for these genes was retrieved from IWGSC RefSeq 1.0 annotation. This led to the identification of 35 high confidence genes predicted to have a plant-disease related function based on a thorough review of the literature (Table 3). In the region spanning QTL *QSnb.sdsu-7A*, we identified three protein-kinase and one receptor kinase domain encoding genes. Similarly, four genes were found in *QSnb.sdsu-2A* region, including two NBS-LRR family proteins encoding genes, that could be used to find the genes for this QTL. The *QSnb.sdsu-6B* region harbored five genes of importance, including an NBS-LRR protein and a receptor-like kinase. The region spanning the fourth QTL, *QSnb.sdsu-2D*, consisted of 14 genes with 12 NBS-LRR domain encoding genes and two genes with a protein-kinase domain. Furthermore, eight putative candidate genes with predicted role in plant defense response were identified in the region covering *QSnb.sdsu-4A*. (Table 3).

**Table 3:** Summary of the candidate genes in the identified QTL regions.

QTL	Chromosome	Gene	Functional annotation
*QSnb.sdsu-2A*	2A	TraesCS2A02G589900	Receptor-like kinase
	2A	TraesCS2A02G590100	Disease resistance protein: NB-ARC
	2A	TraesCS2A02G590200	Disease resistance protein RPM1: NB-ARC
	2A	TraesCS2A02G593500	Receptor kinase, putative
*QSnb.sdsu-2D*	2D	TraesCS2D02G018300	NB-ARC domain-containing disease resistance protein
	2D	TraesCS2D02G018400	NB-ARC domain-containing disease resistance protein
	2D	TraesCS2D02G018500	NB-ARC domain-containing disease resistance protein
	2D	TraesCS2D02G019200	NB-ARC domain-containing disease resistance protein
	2D	TraesCS2D02G019400	NB-ARC domain-containing disease resistance protein
	2D	TraesCS2D02G019500	NB-ARC domain-containing disease resistance protein
	2D	TraesCS2D02G019700	NB-ARC domain-containing disease resistance protein
	2D	TraesCS2D02G019800	Receptor-like protein kinase
	2D	TraesCS2D02G019900	NB-ARC domain-containing disease resistance protein
	2D	TraesCS2D02G020000	NB-ARC domain-containing disease resistance protein
	2D	TraesCS2D02G020300	NB-ARC domain-containing disease resistance protein
	2D	TraesCS2D02G020400	NB-ARC domain-containing disease resistance protein
	2D	TraesCS2D02G020700	NBS-LRR-like resistance protein
	2D	TraesCS2D02G021400	Receptor-like protein kinase
*QSnb.sdsu-4A*	4A	TraesCS4A02G496700	NBS-LRR-like resistance protein
	4A	TraesCS4A02G496800	NBS-LRR-like resistance protein
	4A	TraesCS4A02G497100	Disease resistance protein (TIR-NBS-LRR class) family
	4A	TraesCS4A02G497300	Receptor-like protein kinase
	4A	TraesCS4A02G497800	Receptor kinase 1
	4A	TraesCS4A02G497900	Receptor-like kinase
	4A	TraesCS4A02G499000	Receptor-like kinase
	4A	TraesCS4A02G499400	Receptor-like kinase
*QSnb.sdsu-6B*	6B	TraesCS6B02G050300	NBS-LRR resistance-like protein
	6B	TraesCS6B02G050800	Protein kinase family protein
	6B	TraesCS6B02G050900	Protein kinase family protein
	6B	TraesCS6B02G051000	Protein kinase family protein
	6B	TraesCS6B02G051700	Receptor-kinase, putative
*QSnb.sdsu-7A*	7A	TraesCS7A02G544000	Protein kinase family protein
	7A	TraesCS7A02G544100	Serine/threonine-protein kinase
	7A	TraesCS7A02G544200	Serine/threonine-protein kinase
	7A	TraesCS7A02G544600	Receptor kinase 1

Gene IDs and functional annotation are based on IWGSC CS RefSeq v 1.1 (2018).

## Discussion

SNB is an important fungal disease of wheat and a severe infection can cause significant yield losses. Thus, exploring the resistance sources among the existing germplasm and utilizing them in wheat breeding could be an effective disease management strategy. In this study, we used a US hard winter wheat association-mapping panel (HWWAMP), which turned out to be a good source of SNB resistant germplasm.

**Figure 5:**
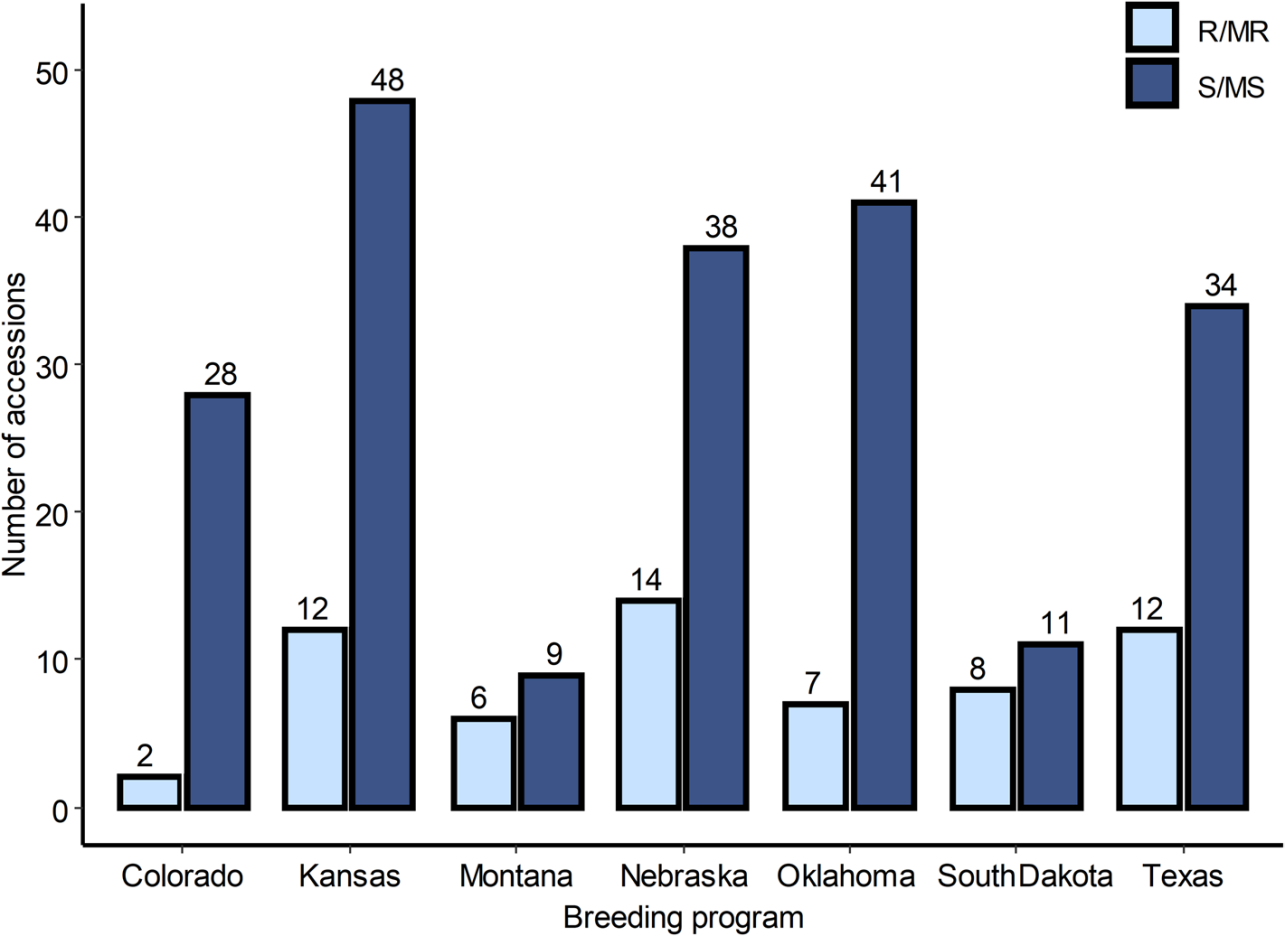
Distribution of the SNB resistance among the 274 accessions of hard winter wheat association mapping panel (HWWAMP) originating from different breeding programs.

A total of 274 accessions of HWWAMP were evaluated for resistance against SNB. Out of 274 lines, 112 (40.87%) lines were identified as resistant or moderately resistant to SNB, indicating novel or existing sources of resistance present in the HWWAMP. Around 50% of tested germplasm, including spring wheat and winter wheat, was found resistant to SNB in the previous studies^36,45,46^. Lines with SNB resistance were present among all breeding programs from Colorado, Kansas, Montana, Nebraska, Oklahoma, South Dakota, and Texas (Fig. 5). Interestingly, most of the breeding programs from where the accessions were collected have developed a few highly resistant germplasms against SNB. However, it is important to note that about 60% of the tested germplasm were susceptible to SNB in this study. One possible explanation for wide susceptibility in the hard winter wheat breeding programs of the central U.S. states is retention of *Tsn1,* which could be a result of deliberate selection of some other resistance gene and/or likely linkage to important agronomic traits^7^.

In addition to SNB screening, we evaluated HWWAMP against three necrotrophic effectors (NEs), namely SnToxA, SnTox1, and SnTox3. The *P. nodorum* isolate Sn2000 contains two important NEs, SnToxA and SnTox1^17,18,29^, that play a significant role in disease development. Out of the 274 accessions, 209 (76%), 15 (5%), and 65 (24%) were found sensitive to SnToxA, SnTox1, and SnTox3, respectively, indicating the higher prevalence of *Tsn1*-SnToxA and *Snn3*-SnTox3 interactions in the germplasm from the Great Plains region. Most of the accessions (76%) in our study were sensitive to SnToxA, owing to the presence of susceptibility gene *Tsn1* in most of the tested material. Sensitivity to SnToxA is regulated by the expression of sensitivity gene *Tsn1*, and happens to be the one with the largest positive effect on susceptibility^16,17^. The frequency of SnToxA sensitive lines varies in different germplasm; for example, only 10% of accessions were found sensitive in a British winter wheat germplasm collection^47^, whereas it was 45% in Scandinavian varieties^30^ and 65% in Western Australian spring wheat^48^. In contrast to SnToxA, 95% of HWWAMP lines (259) were insensitive to SnTox1, suggesting the absence of *Snn1* gene^29^ in most of the accessions. Furthermore, we did not find any significant MTAs between SNPs and SnTox1 sensitivity, indicating a weak *Snn1*-SnTox1 interaction. A few accessions sensitive to SnTox1 in the current study are in line with previous reports in hexaploid wheat^32,47,49^. The frequency of accessions sensitive to SnTox3 (24%) in the current study was similar to that reported in the European germplasm^47^. We could not identified *Snn3* in GWAS analysis for SNB response due to lack of SnTox3 in Sn2000; however, GWAS for SnTox3 sensitivity identified a significant association in the region corresponding to *Snn3* gene suggesting the presence of *Snn3* in the winter wheat panel.

Further, we investigated whether NE sensitivity contributes toward the SNB susceptibility. We found a significant difference (*P* < 2.2e−16) for SNB severity between sensitive and insensitive groups for SnToxA. The insensitive lines were more resistant to SNB than the sensitive lines. No such differences were found between SnTox1 sensitive and insensitive groups (Fig. 1D). Interestingly, most of the highly resistant lines (score; 1–1.1), were insensitive to all the three NEs (Supplementary Table S2) while the highly susceptible lines (score; 4–5) were sensitive to at least one NE, suggesting the NE triggered susceptibility. A significant (*P* < 0.001) correlation was also observed between the effector sensitivity reaction and disease score in a recent study^32^. We also identified six lines that were insensitive to all three NEs (SnToxA, SnTox1, and SnTox3) but susceptible (Score; 3–5) to SNB isolate Sn2000 at the seedling stage, suggesting the possible interactions of other NE with host susceptibility gene(s) or lack of host resistance genes^50^. Necrotrophic effector-triggered susceptibility in the wheat-*P. nodorum* pathosystem is a complicated process and the effects can vary depending on the genetic backgrounds of the pathogen and host^41,51^.

Our GWAS identified significant associations on chromosomes 1B, 2AL, 2DS, 4AL, 5BL, 6BS, and 7AL, representing seven distinct QTLs for SNB resistance/susceptibility. Based on the types of markers used in similar studies^38–40,42^, it is difficult to precisely compare the previously identified regions to those of our study. However, to facilitate the comparison of QTLs reported in other studies with our study, we identified the approximate genomic locations of QTLs on IWGSC RefSeq ver 1.1^52^. In agreement with the previous studies^32,38,39^, the QTL with the largest effect was detected in the region corresponding to the genomic region of *Tsn1*^39^. We identified one QTL (*QSnb.sdsu-2D*) on the short arm of chromosome 2D, which physically maps to 9 Mb on the wheat reference genome. Two recent studies^53,54^ have also reported SNB resistance QTLs in the same region (~ 14–15 Mb) at the adult plant stage. This region also harbors the SnTox2 sensitivity gene *Snn2* (6–12 Mb)^53^; thus, *QSnb.sdsu-2D* identified in this study co-locates with this sensitivity gene.

A robust QTL (*QSnb.sdsu-7A*) was detected on the long arm of chromosome 7A, which physically mapped to the distal portion of the long arm at 721 Mb. Previous studies^32,40,55^ have reported a QTL for seedling resistance on the long arm of chromosome 7A at around 550 Mb and 590 Mb. Therefore, *QSnb.sdsu-7A,* which maps 200 Mb distal, could be a novel QTL on the terminal end of chromosome 7A. Furthermore, we identified a significant association for SNB response on the short arm of chromosome 6B, physically mapping around 30 Mb on the reference genome. Ruud et al.^32^ recently reported an adult plant resistance QTL on the short arm of chromosome 6B, located in the same region (20–47 Mb) on the physical map. Thus, co-location of *QSnb.sdsu-6B* with Ruud et al.^32^ suggests that the same locus may confer resistance at the juvenile and adult plant stage.

In addition to these regions, we identified significant QTLs on chromosomes 1B, 2AL, and 4AL. The QTL identified on chromosome 4AL was physically located at ~ 740 Mb on the reference genome. Liu et al.^40^, also reported an association in a close approximation (~ 710 Mb) from a GWAS using US winter wheat cultivars. Two other mapping studies^41,56^ reported a significant QTL in the same region; however, we could not identify the physical location owing to different types of markers. Most likely, *QSnb.sdsu.4A* corresponds and validates these regions and plays a role in resistance/susceptibility at the seedling stage. The current study also validated another genomic region on chromosome 2AL at ~ 780 Mb. Several studies have identified loci for seedling and adult plant resistance in the same region^20,47,53,54^. In a very recent report, Lin et al.^53^ identified an adult plant resistance QTL in the same region positioned between 755 and 780 Mb, overlapping with the *QSnb.sdsu.2A*.

Further, we analyzed the effect of accumulation of resistant alleles at seven of the detected QTLs to verify the quantitative resistance. Six different genotype groups (group 0 to group 5) were observed carrying ‘zero’ to ‘six’ resistant alleles at identified loci, respectively. As found in earlier reports^41^, accessions with a higher number of resistant alleles (either four or five) exhibited a high level of resistance. We compared the six groups using an FDR-corrected pairwise t-test and found significant differences in the level of resistance to SNB, which explains the additive and complex nature of SNB resistance^14^. In addition, we identified several released cultivars carrying five (‘Pioneer-2180’ and ‘Shocker’) or four (‘Colt’, ‘TAM304’, ‘Darrel’, ‘Hume’) resistance-associated alleles, with high resistance level. These accessions explain the effectiveness of pyramiding effector insensitivity and resistance-associated QTLs for SNB resistance.

Apart from GWAS for SNB response, we performed association analysis for sensitivity to SnToxA, SnTox1, and SnTox3. We identified significant MTAs for SnToxA and SnTox3; however, no association was detected for SnTox1. Liu et al.^40^, also reported similar results from the GWAS employing winter wheat cultivars. The MTAs identified for SnToxA and SnTox3 corresponds to the genomic regions of *Tsn1*^17^ and *Snn3*^30^ genes, respectively. The low variation for SnTox1 sensitivity among HWWAMP accessions could be the potential reason for not detecting any MTAs for SnTox1 sensitivity.

The five genomic regions associated with SNB resistance/susceptibility were screened for candidate genes based on the Chinese Spring reference genome RefSeq v1.1^52^. In wheat, majority of the characterized disease resistance genes encode intracellular immune receptors of the nucleotide binding-site–leucine-rich repeat (NBS-LRR) family, wall-associated kinases (WAKs), receptor-like kinases (RLKs), and protein kinases as the protein product^57^. For instance, *Tsn1*, encodes S/T protein kinase-NLR containing protein^16^. *Snn1* encodes a Wall-associated kinase protein^49^. Similarly, *Stb6* governs resistance against *Septoria tritici* blotch in wheat and belongs to the wall-associated kinase family of proteins^58^. Therefore, these gene families are expected to play a role in the plant defense response. Our study identified several genes encoding NBS-LRR domain, wall-associated kinases, receptor-like kinases, or protein kinases in the regions spanning identified QTLs (Table 3). These disease-related genes could be useful for the identification of potential candidates responsible for resistance/susceptibility to SNB.

In summary, we identified and validated several QTLs for SNB resistance/susceptibility in hard winter wheat. These QTLs could be easily employed in breeding programs using the associated markers to improve the SNB resistance in wheat. The comparison of groups carrying a different number of resistant/susceptibility alleles suggests the additive nature of SNB resistance. Thus, stacking of identified resistance-associated QTLs and known effector insensitivity genes could help in developing SNB resistant cultivars. The highly resistant winter wheat accessions (‘Pioneer-2180’ and ‘Shocker’) with up to five favorable alleles could be valuable germplasm for the wheat breeders. These accessions can be further evaluated against other prevalent isolates and for adult-plant resistance; and used in the breeding programs to improve SNB resistance.

## Methods

### Plant materials

We used a set of 274 lines, selected from a hard winter wheat association mapping panel (HWWAMP) consisting of 299 accessions developed under the USDA Triticeae Coordinated Agricultural Project (TCAP)^43^. The association mapping panel comprises released varieties and breeding lines from the US Great Plains region, including Colorado, Kansas, Montana, Nebraska, North Dakota, Oklahoma, South Dakota, and Texas. Additional details about the HWWAMP accessions are available in the T3/Wheat database (https://triticeaetoolbox.org/wheat/). Two differential lines, Salamouni (resistant to SNB) and Glenlea (susceptible to SNB), were included as checks for *Stagonospora nodorum* blotch (SNB) evaluations. The plant material used in this study did not require any permission/license for evaluation and all the necessary guidelines were followed.

### Evaluations for seedling resistance to SNB

A set of 274 lines, along with two differential lines (Salamouni-SNB resistant and Glenlea-susceptible) were evaluated for Stagonospora nodorum blotch (SNB) reaction caused by *Parastagonospora nodorum* (isolate Sn2000) at the seedling stage under greenhouse conditions in three independent experiments. The *Paratagonospora nodorum* isolate Sn2000 is reported to produce at least two host-selective toxins, SnTox1 and SnToxA^17^. In each of the experiments, all the lines were planted in a cone trainer (RAYLEACH “CONE TRAINER” Single-Cell System) filled with SUNSHINE R 360 potting mixture (SUNGRO HORTICULTURE, Agawam, MA, USA), with three plants per cone. The cones were placed in racks (Stuewe & Sons, Tangent, OR, USA) in a completely randomized design with three biological replicates.

A pure culture of isolate Sn2000 was grown on plates containing V8PDA medium and incubated at 21 °C under light for 7 days. The plates were flushed with 30 mL sterile distilled water followed by scraping with a sterile glass slide to collect the pycnidiospores. The inoculum concentration was estimated using a hemocytometer and the final concentration was adjusted to 1 × 10^6^ mL^−1^ before inoculation.

Seedlings were spray inoculated at the two-leaf stage in the greenhouse using the previously described method^59^ and placed in a humidity chamber with 100% humidity for 24 h to enhance the infection process. Thereafter, the plants were moved back to the greenhouse bench. Eight days after inoculation, the disease reactions were scored using a numerical scale of 0 to 5 based on the lesion type^29^, where 0 = absence of visible lesions (highly resistant); 1 = few penetration points, with lesions consisting of flecking or small dark spots (resistant); and 5 = large coalescent lesions with very little green tissue remaining (highly susceptible).

### Infiltrations with NEs

All the 274 accessions along with the differential checks were grown as described above in three independent sets for infiltrations with toxins SnToxA, SnTox1, and SnTox3, respectively. Three fully expanded leaves of each accession were infiltrated with SnToxA, SnTox1, and SnTox3 culture filtrates using a needle-less syringe following the methodology of Faris et al.^60^. Dr. Timothy Friesen, USDA-ARS, Fargo, ND, kindly provided all the three NEs culture filtrates. Leaves of the seedlings were infiltrated with the equal volume (20–25 μl) of the filtrate. After 72 h of infiltration, the seedlings were rated for infiltration responses. The sensitivity reactions were scored as sensitive = necrosis and tissue collapse; or insensitive = no reaction/necrosis.

### Statistical analysis

The linear mixed model (LMM) approach was used to analyze the phenotypic data for SNB inoculations, considering all factors as random. The data was analyzed based on the following model:

$${\text{Y}}_{{{\text{ijk}}}} = \mu + {\text{G}}_{{\text{i}}} + {\text{E}}_{{\text{j}}} + {\text{GE}}_{{{\text{ij}}}} + {\text{R}}_{{{\text{i}}({\text{j}})}} + {\text{e}}_{{{\text{ijk}}}}$$

where “µ” stands for the population mean, “G_i_” stands for genotypes, “E_j_” for experiments, “R_i(j)_” for replications nested under experiments, and “e_ijk_” for the random error. The analysis was performed in the R environment^61^. Correlation between effector sensitivity and SNB score was estimated in R. Different groups carrying the different number of resistant alleles were compared for allele stacking analysis using pairwise t-test with FDR^62^ correction in R.

### SNP genotyping

The HWWAMP was genotyped using the wheat INFINIUM 90K iSelect array (ILLUMINA Inc. San Diego, CA) under the USDA-TCAP^63^ yielding a total of 21,555 SNPs. The genotypic data is publicly available and was obtained from the T3 Toolbox (https://triticeaetoolbox.org/wheat/genotyping/display_genotype.php?trial_code=TCAP90K_HWWAMP). As a quality control, the genotypic data were filtered with a minimum allele frequency (MAF) < 0.05 and more than 10% missing SNP data, leaving 15,590 SNP markers, which were used for further analysis. The genetic positions of the wheat INFINIUM 90K iSelect SNP markers were obtained from the consensus genetic map of 46,977 SNPs^64^. The physical positions of the SNPs with significant associations with SNB response were obtained by ‘blastn’ searching the flanking sequences of respective SNPs to wheat Chinese Spring RefSeq v1.1 assembly^52^.

### Population structure and linkage disequilibrium

Population structure within the 274 HWWAMP accessions was inferred using a model-based Bayesian cluster analysis program, STRUCTURE v2.3.4^65^ to estimate the number of sub-populations. The admixture model was used with the number of assumed groups set from k = 1 to 10. The analysis was performed in five independent replicates, with 10,000 burn-in replicates and 10,000 Markov Chain Monte Carlo (MCMC) iterations in each of the runs. Structure Harvester^66^ was used to infer the optimum number of clusters using statistic ΔK (delta K)^67^, which is based on the rate of change in the log probability of given data, between successive K values. The structure bar plot for the optimum number of clusters was drawn using Structure Plot v2.0^68^. Linkage disequilibrium (LD) for the HWWAMP was analyzed using TASSEL v5.0^69^ with only 1,842 markers, taking out non-informative markers in our previous study^35^. The LD decay distances for the whole, as well as individual genomes, was estimated by plotting the r^2^ values against the genetic distance (cM) between the markers.

### Association mapping for SNB and NEs

Association analysis was performed using two different algorithms to select the model that better fits the data. The first was the MLM algorithm (with optimum compression and P3D), a single locus method^70^, implemented in TASSEL (Trait Analysis by association, Evolution, and Linkage) v 5.0 software^69^. The second model was FarmCPU (fixed and random model circulating probability unification)^71^, a multilocus method implemented through Genomic Association and Prediction Integrated Tool (GAPIT)^72^ in the R environment. Both of the two models took into account a K-PC model^70^, by including the kinship and population structure as covariates to improve the statistical power of association analysis. Kinship (K) was estimated using the Centered IBS (identity by state) method^73^. The first three Q-variates obtained through STRUCTURE analysis were used as covariates in the models.

Generally, MLM is used as it incorporates kinship and population structure as covariates to minimize the confounding effects and controls the false positives. However, it leads to several false negatives due to the confounding between population structure and quantitative trait nucleotides (QTNs). We evaluated FarmCPU, an improved multiple-locus model (testing multiple markers simultaneously) that further eliminates the drawbacks of the MLM algorithm by using associated markers as covariates to perform marker tests within a fixed-effect model. Further, it employs a separate random effect model to optimize the association between tested markers and the trait^71^.

These two algorithms were compared using the quantile–quantile (QQ) plots obtained from the analysis. The QQ plots suggested that FarmCPU performed better than the MLM algorithm for Stagonospora (*syn*. Septoria) nodorum blotch (isolate Sn2000) response data. Therefore, we used FarmCPU to detect the MTAs and identify candidate genes for SNB resistance using the grand mean of disease score from three independent experiments. The MLM algorithm fits better on the effector infiltration data. Thus, we used the best model to report the MTAs for each trait. The threshold for significance was corrected for multiple testing using a Bonferroni correction and False Discovery Rate (FDR) correction^62^. Associations surpassing the corrected *p*-value were declared as significant MTAs.

Allele stacking analysis was performed to study the accumulation effect of alleles associated with SNB resistance. The accessions from the HWWAMP were grouped based on the number of resistant alleles present in them. These groups were compared using FDR corrected pairwise t-test to verify the additive effect of the resistant alleles on SNB reaction.

### Identification of candidate genes

The physical positions of all significant SNPs on Chinese spring (CS) RefSeq v1.1 were obtained from the IWGSC database by BLASTN searching the flanking sequences of respective SNPs^52^. The gene models within ± 2 Mb of the most significant associated marker were derived from IWGSC RefSeq 1.1. The high confidence genes in the selected region were retrieved and IWGSC Functional Annotation v1.0^52^ was used to identify the genes with putative disease resistance functions based on a thorough review of the literature.

## Supplementary Information

Supplementary Information.

## Data Availability

All datasets generated for this study are included in the article/Supplementary Material. Additional physiological and agronomic data about the HWWAMP accessions is available in the T3/Wheat database (https://triticeaetoolbox.org/wheat/pedigree/pedigree_info.php).
