# Supplementary material for: Genome-wide association analysis permits characterization of Stagonospora nodorum blotch (SNB) resistance in hard winter wheat
Source: Sci Rep. 2021 Jun 15;11:12570. doi: 10.1038/s41598-021-91515-6 (PMC8206080; doi:10.1038/s41598-021-91515-6)
Supplement: Supplementary file 1 — Supplementary Information. [file 41598_2021_91515_MOESM1_ESM.pdf]

Supplementary Material

Genome-wide association analysis permits characterization of Stagonospora nodorum blotch (SNB) resistance in hard winter wheat

Rami AlTameemi[1$], Harsimardeep S. Gill[1$], Shaukat Ali[1], Girma Ayana[1], Jyotirmoy Halder[1], Jagdeep S. Sidhu[1], Upinder S. Gill[2], Brent Turnipseed[1], Jose L. Gonzalez Hernandez [1], Sunish K. Sehgal[1]*

Supplementary Figure S1. Structure analysis for the 274 accessions of hard winter wheat association mapping panel (HWWAMP). **(A)** Structure plot showing three subpopulations at K = 3. **(B)** Pie chart representing the number of accessions in each of the subpopulations. **(C**) the Delta K estimate for differing number of subpopulations (k).

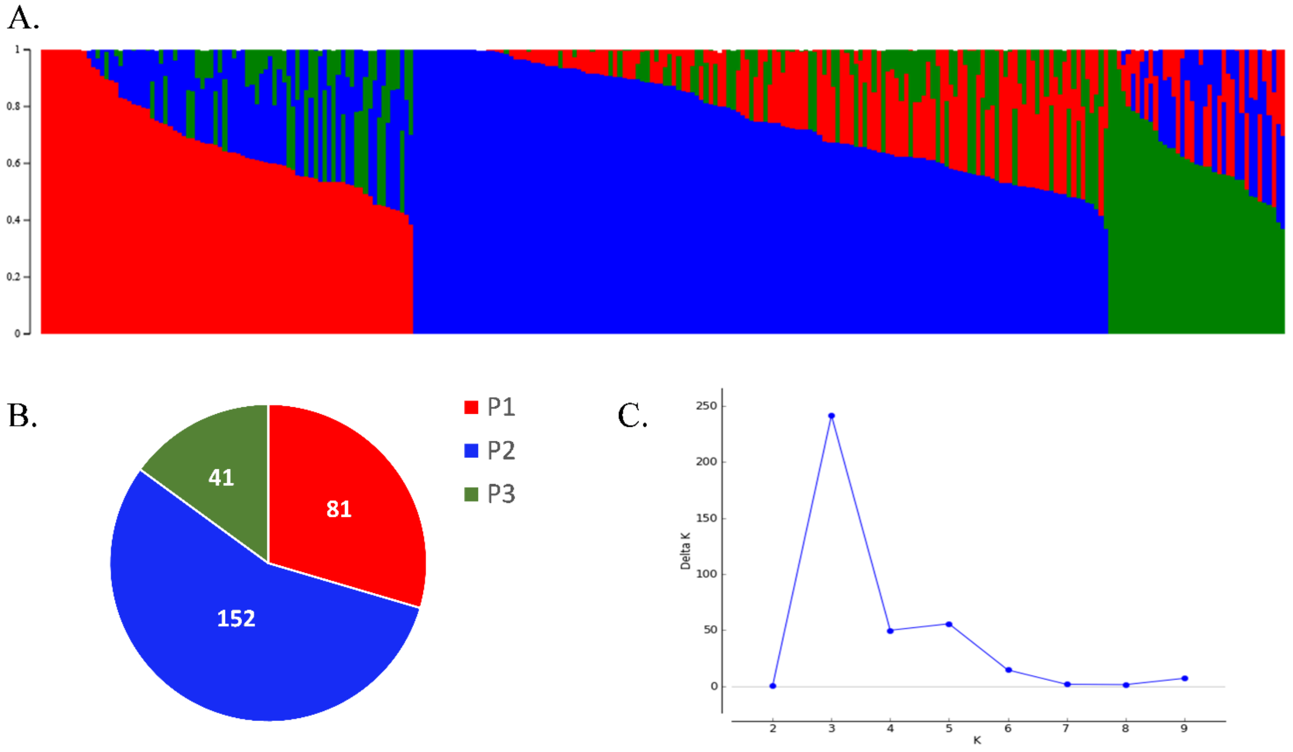

Supplementary Figure S2. Quantile-quantile (QQ) plots of association analysis for different traits. (a) MLM model fitted on SNB infection data; (b) FarmCPU model fitted on SNB infection data; (c) MLM model fitted on SnToxA infiltration data; (d) FarmCPU model fitted on SnToxA infiltration data; (e) MLM model fitted on *SnTox3* infiltration data; and (f) FarmCPU model fitted on *SnTox3* infiltration data

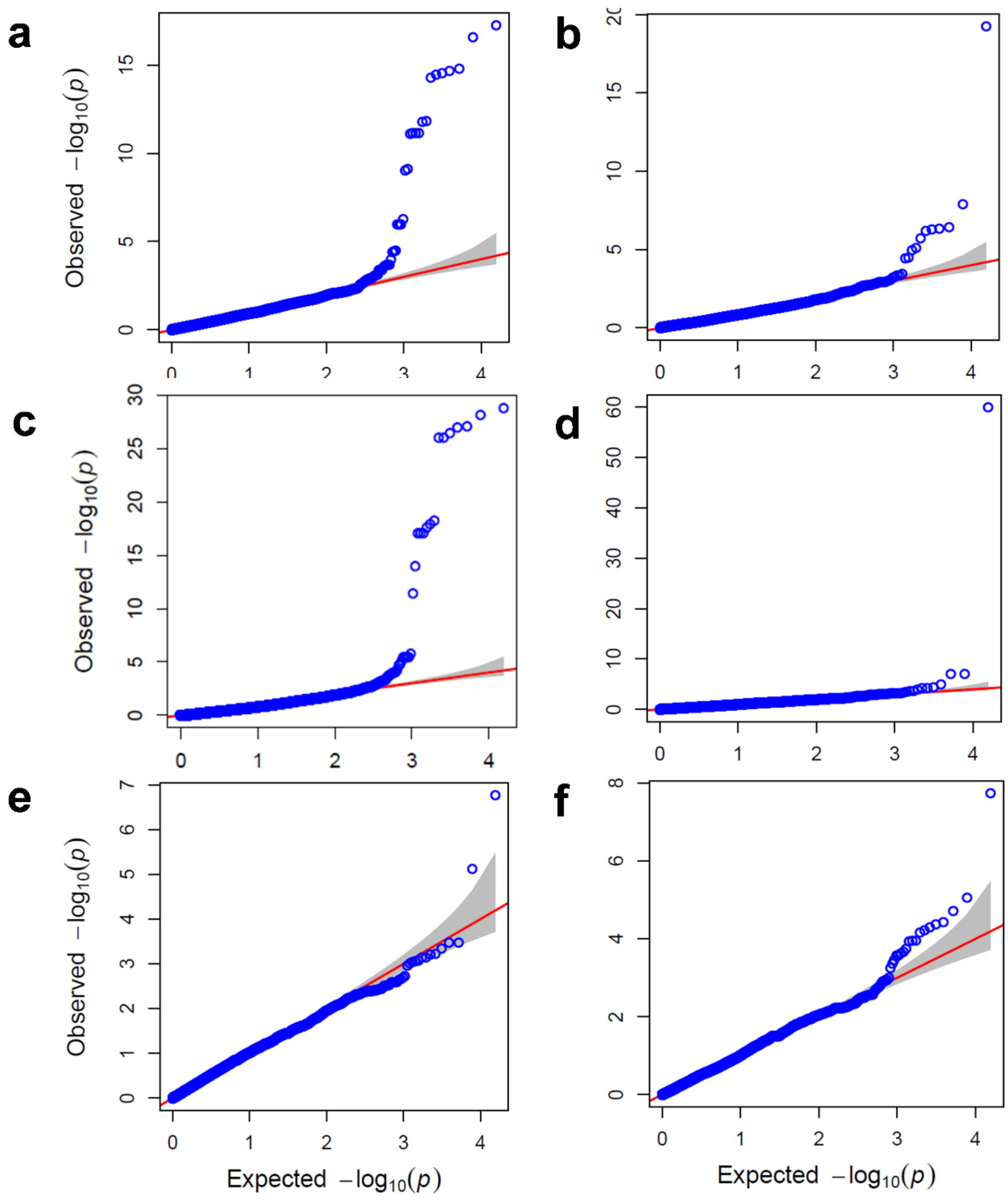

Supplementary Table S1. Grand mean for SNB responses (0-5 scale) and Necrotrophic Effectors (NEs) reaction evaluated on 274 accessions of hard winter wheat association mapping panel (HWWAMP). The NEs sensitivity was recorded as I (Insensitive) or S (Sensitive) reaction.

Accession	SNB Response	SnToxA Reaction	SnTox1 Reaction	SnTox3 Reaction
2180	1	I	I	I
2145	2.777777778	S	I	S
2174-05	3.222222222	S	I	I
ABOVE	2.333333333	S	I	I
AKRON	3.333333333	S	S	I
ALICE	1.888888889	I	I	S
ALLIANCE	1.333333333	I	I	I
ANTELOPE	3.555555556	S	I	I
ANTON	1.222222222	S	I	S
ARAPAHOE	3.777777778	S	I	I
ARLIN	2.222222222	I	I	S
AVALANCHE	2	I	I	S
BAKERS_WHITE	2	S	I	I
BENNETT	2.444444444	I	I	I
BIG_SKY	2.777777778	I	I	I
BILL_BROWN	3.111111111	I	I	S
BILLINGS	1.444444444	I	I	I
BISON	2.333333333	S	I	S
BOND_CL	3	S	S	S
BUCKSKIN	3.333333333	S	I	I
BURCHETT	4	S	I	I
BYRD	2.222222222	S	I	I
CAMELOT	2	I	I	S
CAPROCK	2.777777778	S	I	I
CARSON	3	S	I	I
CENTERFIELD	3.111111111	S	I	I
CENTURK78	2.555555556	S	I	I
CENTURY	1.333333333	I	I	S
CHENEY	4.777777778	S	I	I
CHEYENNE	3.888888889	S	I	I
CHISHOLM	4	S	I	S
CO03064	2.444444444	S	I	I
CO03W043	2.333333333	S	S	S
CO04025	4	S	I	I

CO04393	3.333333333	I	I	S
CO04499	3.444444444	S	I	S
CO07W245	3.111111111	S	I	S
CO940610	2.777777778	S	I	I
COLT	1	I	I	S
COMANCHE	3.666666667	S	I	I
COSSACK	3	I	I	I
COUGAR	3.555555556	S	I	I
CREST	2.555555556	S	I	I
CRIMSON	2	I	I	S
CULVER	4.111111111	S	I	I
CUSTER	4	S	I	I
CUTTER	2	S	S	I
DANBY	3	S	I	I
DARRELL	1.555555556	I	I	I
DAWN	4.111111111	S	I	I
DECADE	1.333333333	I	I	I
DENALI	3.666666667	S	I	I
DODGE	3.666666667	S	I	I
DUSTER	4	S	II	I
E2041	1	I	I	I
EAGLE	4.444444444	S	I	I
ENHANCER	3.777777778	S	I	I
EXPEDITION	4	S	I	I
FULLER	3.444444444	S	I	I
G1878	3.222222222	S	I	I
GALLAGHER	3.555555556	S	I	I
GARRISON	2	S	I	I
GENOU	1.111111111	I	I	I
GENT	3.111111111	S	I	I
GOODSTREAK	4.222222222	S	I	I
GUYMON	3.222222222	S	I	I
HAIL	4	S	S	I
HALLAM	4	S	I	I
HALT	2.666666667	S	S	S
HARDING	4	S	I	I
HARRY	3.333333333	S	I	I
HATCHER	2.333333333	S	I	I
HEYNE	3.555555556	S	I	S
HG-9	4	S	I	I
HOMESTEAD	3.888888889	S	I	I

HONDO	3.333333333	S	I	S
HUME	1.666666667	I	I	I
HV906-865	2.666666667	S	I	I
HV9W03-1379R	3.333333333	S	I	S
HV9W03-1551WP	2.333333333	I	I	I
HV9W03-1596R	1.333333333	I	I	I
HV9W05-1280R	2	S	I	I
INFINITY_CL	3	S	I	S
INTRADA	2.333333333	S	I	I
JAGALENE	3.888888889	S	I	S
JAGGER	2	S	I	I
JERRY	4.666666667	S	I	I
JUDEE	4.222222222	S	I	S
JUDITH	3.333333333	S	I	I
JULES	4	S	S	I
KARL_92	3.222222222	S	I	I
KAW61	4	S	I	I
KEOTA	3.333333333	S	I	I
KHARKOF	3.888888889	S	S	I
KIOWA	4.222222222	S	I	I
KIRWIN	3.666666667	S	I	I
KS00F5-20-3	2	S	I	S
LAKIN	3.888888889	S	I	S
LAMAR	3.111111111	S	I	I
LANCER	3.777777778	S	I	I
LARNED	4.111111111	S	I	I
LINDON	3.444444444	S	I	I
LONGHORN	4.444444444	S	I	I
MACE	2	S	I	S
MCGILL	3.777777778	S	I	I
MILLENNIUM	1.888888889	I	I	I
MIT	1.666666667	S	I	I
MT0495	2	S	I	I
MT06103	1.777777778	I	I	I
MT85200	2.888888889	S	I	I
MT9904	1.666666667	I	I	I
MT9982	3.555555556	S	I	I
NE02558	2	S	I	I
NE04490	2	S	I	I
NE05430	2.555555556	S	I	I
NE05496	2.333333333	I	I	I

NE05548	2.666666667	S	I	I
NE06545	1.222222222	I	I	I
NE06607	2.777777778	S	I	I
NE99495	3.333333333	I	I	S
NEKOTA	1.111111111	I	I	I
NELL	1.222222222	I	I	I
NEOSHO	2.222222222	S	I	I
NEWTON	3.666666667	S	I	I
NI06736	3	S	I	I
NI06737	2.111111111	S	I	I
NI07703	3.444444444	S	I	I
NI08707	2.888888889	S	I	I
NI08708	2	S	I	S
NIOBRARA	4	S	I	I
NORKAN	4.333333333	S	I	I
NORRIS	3.444444444	S	I	I
NUFRONTIER	1.555555556	I	I	N
NUHORIZON	1.888888889	I	I	S
NUPLAINS	3.222222222	S	I	I
NUSKY	2	S	I	I
NW03666	1.444444444	I	I	I
OGALLALA	3	S	I	I
OK_RISING	4	S	S	I
OK02405	3.888888889	S	I	S
OK04111	4	S	I	I
OK04415	3.333333333	S	I	I
OK04507	2.222222222	S	I	I
OK04525	3.333333333	S	S	I
OK05511	2.555555556	S	I	S
OK05134	4	S	I	I
OK05204	3.444444444	S	I	S
OK05303	3.888888889	S	I	I
OK05312	2.222222222	S	I	I
OK05526	3	S	I	I
OK05711W	3.111111111	S	I	I
OK05723W	1.111111111	I	S	S
OK05830	2.777777778	I	I	I
OK06114	2.111111111	S	S	I
OK06210	2.777777778	I	I	I
OK06318	3.222222222	S	I	I
OK06319	2.222222222	S	I	S

OK06336	4	S	I	S
OK07231	3.555555556	I	I	I
OK07S117	2	I	S	S
OK08328	2.555555556	I	I	I
OK09634	1	I	I	I
OK101	1.333333333	I	I	I
OK10119	2.444444444	S	I	I
OK102	4	S	I	I
OK1067071	3.333333333	S	I	S
OK1067274	3	I	I	I
OK1068002	4	S	I	I
OK1068009	3.444444444	S	I	I
OK1068026	4	S	I	I
OK1068112	3.111111111	S	I	I
OK1070267	3.111111111	I	I	S
OK1070275	2.555555556	S	I	S
ONAGA	2.111111111	S	I	I
OVERLAND	3.444444444	S	I	I
OVERLEY	2.222222222	S	I	I
PARKER	4.333333333	S	I	I
PARKER76	4.888888889	S	I	I
PETE	4	S	I	I
PLATTE	3.555555556	S	I	S
POSTROCK	2.444444444	S	I	I
PRAIRIE_RED	3.222222222	S	I	S
PRONGHORN	4.555555556	S	I	I
PROWERS	3.222222222	S	I	I
RAWHIDE	4	S	I	I
REDLAND	4	S	I	I
RIPPER	3.111111111	S	I	S
RITA	2.888888889	S	S	S
ROBIDOUX	3.555555556	S	I	I
RONL	3.555555556	S	I	I
ROSE	2	I	I	I
ROSEBUD	NA+B278:D280	S	I	I
SAGE	4.444444444	S	I	I
SANDY	3.222222222	S	I	I
SANTA_FE	2.777777778	S	I	I
SCOUT66	3.666666667	S	S	I
SD00111-9	3	S	I	I
SD01058	3.222222222	S	I	I

SD01237	3.222222222	S	I	I
SD05118	3.444444444	I	I	I
SD05210	1	I	I	I
SD05W018	4.111111111	S	I	I
SETTLER_CL	3	S	I	I
SHAWNEE	4	I	I	I
SHOCKER	1.444444444	I	I	I
SIOUXLAND	2.111111111	S	I	I
SMOKYHILL	1.888888889	S	I	I
SPARTAN	4.111111111	S	I	I
STANTON	3	I	I	S
STURDY	3.222222222	S	I	I
STURDY_2K	1	I	I	I
TAM105	3.111111111	S	I	I
TAM107	4	S	I	I
TAM109	2.333333333	S	I	I
TAM110	4	S	I	S
TAM111	3.333333333	S	I	S
TAM112	3.444444444	S	I	I
TAM202	2.222222222	S	I	I
TAM203	1.222222222	S	I	I
TAM303	3.111111111	S	I	S
TAM304	1	I	I	I
TAM400	1.777777778	S	N	N
TAM401	1.666666667	I	I	I
TAMW-101	2.555555556	S	I	I
TANDEM	1.222222222	I	I	I
TARKIO	3.555555556	S	I	I
TASCOSA	4	I	I	S
THUNDER_CL	3.333333333	S	I	S
THUNDERBOLT	3	S	I	S
TREGO	2	I	I	I
TRISON	3.333333333	S	I	I
TRIUMPH64	2.333333333	I	I	S
TURKEY_NEBSEL	2.444444444	S	I	I
TX00V1131	1.666666667	S	I	I
TX01A5936	2.444444444	S	I	I
TX01M5009-28	1.222222222	I	I	I
TX01V5134RC-3	3.444444444	S	I	I
TX02A0252	4	S	I	I
TX03A0148	4	S	I	S

TX03A0563	3.111111111	S	I	S
TX04A001246	2	I	I	S
TX04M410211	1.777777778	S	I	I
TX04V075080	3	S	I	I
TX05A001188	4	S	I	I
TX05A001822	3.666666667	S	I	I
TX05V7259	4.444444444	S	I	S
TX05V7269	4.666666667	S	I	I
TX06A001132	4.444444444	S	I	I
TX06A001281	3	S	I	I
TX06A001386	4	S	I	I
TX06V7266	4	I	I	I
TX07A001279	1.555555556	I	I	S
TX07A001318	4.111111111	S	I	S
TX07A001420	3.666666667	S	I	S
TX86A5606	5	S	I	I
TX86A6880	3	S	I	I
TX86A8072	3.555555556	S	I	S
TX96D1073	1.888888889	S	I	S
TX99A0153-1	3.333333333	S	I	I
TX99U8618	2.444444444	I	I	I
VENANGO	3.222222222	S	I	S
VISTA	4	S	I	I
VONA	3.444444444	S	I	I
W04-417	2.888888889	S	I	S
WAHOO	3.888888889	S	I	I
WARRIOR	3.666666667	S	I	I
WB411W	3.222222222	I	I	I
WENDY	3.444444444	S	I	S
WESLEY	1.222222222	I	I	I
WICHITA	4.222222222	S	I	I
WINDSTAR	1.222222222	I	I	I
WINOKA	2.222222222	S	I	I
YELLOWSTONE	4.222222222	S	I	I
YUMAR	3	S	I	I

Supplementary Table S2. The sensitivity reaction to three Necrotrophic Effectors (NEs) for the HWWAMP accessions exhibiting a high level of resistance against SNB.

Accession	Necrotrophic Effector (NE) sensitivity			SNB score
	SnToxA	SnTox1	SnTox3	
Pioneer-2180	Insensitive	Insensitive	Insensitive	1.0
STURDY-2-K	Insensitive	Insensitive	Insensitive	1.0
Colt	Insensitive	Insensitive	Sensitive	1.0
TAM304	Insensitive	Insensitive	Insensitive	1.0
E2041	Insensitive	Insensitive	Insensitive	1.0
OK09634	Insensitive	Insensitive	Insensitive	1.0
SD05210	Insensitive	Insensitive	Insensitive	1.0
NEKOTA	Insensitive	Insensitive	Insensitive	1.1
Shocker	Insensitive	Insensitive	Insensitive	1.4
Darrel	Insensitive	Insensitive	Insensitive	1.5
